# A case of MRI-induced headache caused by an intracranial foreign body

**DOI:** 10.1093/bjrcr/uaae030

**Published:** 2024-08-29

**Authors:** Hiroyuki Tokue, Azusa Tokue, Yoshito Tsushima

**Affiliations:** Department of Diagnostic and Interventional Radiology, Gunma University Hospital, Maebashi, Gunma 371-8511, Japan; Department of Diagnostic and Interventional Radiology, Gunma University Hospital, Maebashi, Gunma 371-8511, Japan; Department of Diagnostic and Interventional Radiology, Gunma University Hospital, Maebashi, Gunma 371-8511, Japan

**Keywords:** intracranial foreign body, headache, nail gun, MRI

## Abstract

This case report delves into a unique occurrence of MRI-induced headaches attributed to an unsuspected intracranial foreign body. A male patient, presenting persistent headaches, experienced exacerbation of pain upon entering the MRI suite, hindering the imaging procedure. A subsequent head CT scan revealed a nail within the cranial cavity, stemming from a previous nail gun injury. Surgical removal was deemed unsafe, leading to continued observation. This case emphasizes the need for cautious exploration of abnormal symptoms in the MRI suite, urging healthcare professionals to consider potential foreign bodies. The incident underscores the risk of metallic fragments causing complications during MRI procedures and highlights the importance of thorough patient assessment before resorting to MRI imaging.

## Introduction

In contemporary medicine, MRI is an indispensable, noninvasive, and highly sensitive diagnostic tool for neurological assessment. However, patients with unusual symptoms within the confines of an MRI suite present potential challenges that warrant further investigation. This report describes the case of a patient who developed anomalous symptoms in an MRI room and sheds light on the impact of foreign bodies and the required clinical approach. In addition, it highlights the importance of recognizing unexpected intracranial foreign bodies before performing MRI.

## Clinical presentation

A male patient in his 60s presented to our hospital with a headache that had persisted for several months. The patient had no significant medical or surgical history and was conscious, alert, and cooperative. Blood test results and neurological findings were normal. No other specific symptoms apart from the headache were reported. An attempt to conduct a head MRI for further investigation into the cause of the headaches was thwarted, as the patient experienced sudden exacerbation of pain upon entering the 3-T MRI suite. The patient reported that this was not the first time that this peculiar phenomenon had happened to him; 3 years earlier, his headache had similarly worsened when he entered the MRI suite to undergo a lumbar MRI for back pain.

## Investigations

**Figure 1.**
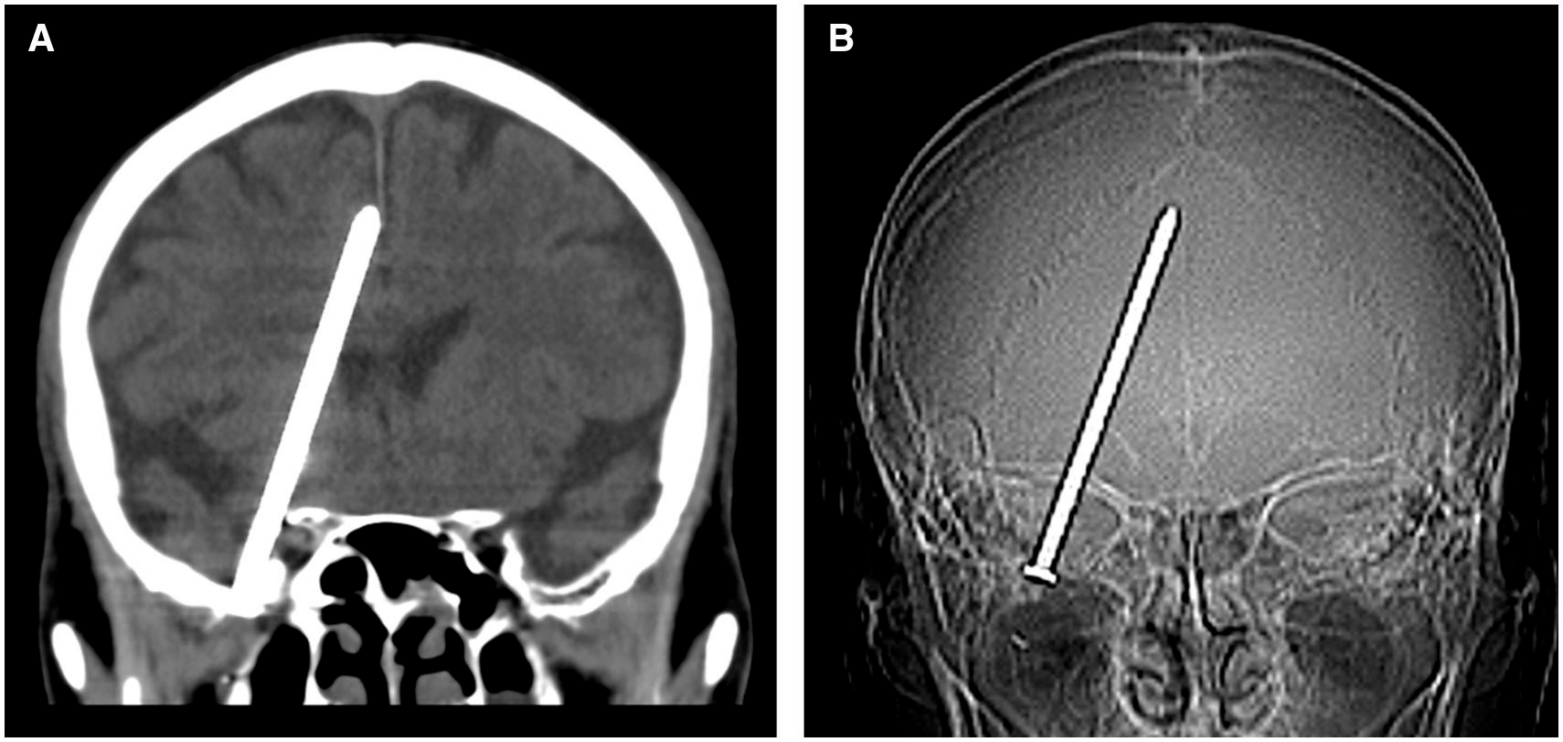
CT shows a nail inside the patient’s cranial cavity. (A) Coronal CT image and (B) CT scout image.

Considering the unusual symptoms in the MRI suite, a decision was made to opt for head CT instead of MRI. CT revealed an unexpected foreign object in the cranial cavity. An approximately 9-cm long nail was observed in the middle cranial fossa lateral to the right orbit, and the tip of the nail was located in the frontal lobe (Figure 1A and B). However, no haemorrhage, granulation, or oedematous changes were observed around the nail.

## Treatment and outcome

Subsequent inquiry into the patient’s medical history revealed a crucial episode: the patient, who was a professional carpenter, experienced profuse bleeding from the nose while using a nail gun 5 years prior. The bleeding may have been caused by partial damage to the paranasal sinuses during the injury. At that time, bleeding from the nose stopped immediately. The patient may have believed the injury was minor or superficial, and the absence of immediate, severe symptoms could have led him to delay further evaluation; therefore, the cause of bleeding was not investigated. This event, unbeknownst at the time, left a magnetic foreign body in the cranial space, which manifested as a source of headaches. After further examination, surgical removal of the intracranial foreign body was deemed unsafe, and the patient remained under observation. Since then, he has experienced occasional headaches; however, the pain has been controlled with nonsteroidal anti-inflammatory drugs.

## Discussion

This case highlights a vital lesson: when confronted with abnormal symptoms in the MRI suite, prudent exploration of the underlying cause supersedes insistence on performing MRI. Furthermore, both physicians and radiological technologists should exercise heightened vigilance during MRI procedures considering the potential presence of such latent anomalies.

Cases of sewing needles and other foreign objects asymptomatically retained in the brain for long periods have been previously reported. Furthermore, skull and brain injuries with a range of penetrating objects, such as blades, nails, pencils, wood splinters, and wires, have been documented.^1^

Since the invention of the nail gun in 1959, it has been commonly used in the construction industry and is easily accessible to the public.^2^ The incidence of nail gun-induced penetrating brain injury has increased with the widespread use of this tool.^2^

In clinical practice, physicians often use MRI to monitor severe headache. However, in this patient, a head MRI performed without careful consideration of the situation could have been fatal. Therefore, physicians should carefully and systematically assess the patient’s pain including the patient’s medical history, symptoms, and potential risk factors before rushing into ordering an MRI. Intracranial foreign bodies, such as metallic fragments, are frequent causes of complications during MRI, as illustrated in this case. Understanding headache in such cases is often difficult. In the case of a sewing needle in the brain, researchers concluded that headaches might be linked to the chemical makeup of rust around the foreign body.^3^ Whether arising from surgical procedures, industrial incidents, or accidents, metallic fragments can become silent disruptors in the magnetic resonance environment. Heightened awareness and prescreening for metallic objects before MRI have emerged as critical considerations.

To the best of our knowledge, MRI-induced headache due to intracranial foreign bodies has not been reported previously. The metal fragment, a remnant of the nail-gun incident, might have undergone physical movement or vibrations within the magnetic field, triggering disturbances in the surrounding neural tissues. This may explain the sudden aggravation of headache experienced by the patient. This physiological interplay underscores the importance of recognizing the potential impact of metallic foreign bodies on MRI.

A consensus on whether needles left after surgery should be surgically removed is lacking.^4^^,^^5^ The patient in the present case is being followed up for the following reasons. First, the nail was not the main problem and was discovered incidentally during the investigation of headache. Second, the potential surgical damage could have had a negative impact on the patient’s quality of life.

## Learning points

- This report highlights the importance of a careful approach by healthcare professionals to unusual symptoms in the MRI room and the importance of communication with the patient. Any unusual symptoms in the MRI room should be carefully evaluated to determine the cause, and physicians performing the MRI should consider the presence of a foreign body.
- We underscore the critical importance of thorough pre-MRI assessment procedures, especially in ensuring patient safety in the presence of potential metallic foreign bodies. Such vigilance can prevent severe outcomes and reinforce the essential role of safety protocols in MRI procedures.
